# Design and Test of a Soil Profile Moisture Sensor Based on Sensitive Soil Layers

**DOI:** 10.3390/s18051648

**Published:** 2018-05-21

**Authors:** Zhenran Gao, Yan Zhu, Cheng Liu, Hongzhou Qian, Weixing Cao, Jun Ni

**Affiliations:** National Engineering and Technology Center for Information Agriculture, Jiangsu Key Laboratory for Information Agriculture, Key Laboratory for Crop System Analysis and Decision Making, Ministry of Agriculture, Jiangsu Key Laboratory for Information Agriculture, Jiangsu Collaborative Innovation Center for the Technology and Application of Internet of Things, Nanjing Agricultural University, Nanjing 210095, China; gzrsunrise@163.com (Z.G.); yanzhu@njau.edu.cn (Y.Z.); lliuchemg@163.com (C.L.); zddlth@163.com (H.Q.)

**Keywords:** soil profile, high-frequency capacitance, moisture sensor, field test

## Abstract

To meet the demand of intelligent irrigation for accurate moisture sensing in the soil vertical profile, a soil profile moisture sensor was designed based on the principle of high-frequency capacitance. The sensor consists of five groups of sensing probes, a data processor, and some accessory components. Low-resistivity copper rings were used as components of the sensing probes. Composable simulation of the sensor’s sensing probes was carried out using a high-frequency structure simulator. According to the effective radiation range of electric field intensity, width and spacing of copper ring were set to 30 mm and 40 mm, respectively. A parallel resonance circuit of voltage-controlled oscillator and high-frequency inductance-capacitance (LC) was designed for signal frequency division and conditioning. A data processor was used to process moisture-related frequency signals for soil profile moisture sensing. The sensor was able to detect real-time soil moisture at the depths of 20, 30, and 50 cm and conduct online inversion of moisture in the soil layer between 0–100 cm. According to the calibration results, the degree of fitting (*R*^2^) between the sensor’s measuring frequency and the volumetric moisture content of soil sample was 0.99 and the relative error of the sensor consistency test was 0–1.17%. Field tests in different loam soils showed that measured soil moisture from our sensor reproduced the observed soil moisture dynamic well, with an *R*^2^ of 0.96 and a root mean square error of 0.04. In a sensor accuracy test, the *R*^2^ between the measured value of the proposed sensor and that of the Diviner2000 portable soil moisture monitoring system was higher than 0.85, with a relative error smaller than 5%. The *R*^2^ between measured values and inversed soil moisture values for other soil layers were consistently higher than 0.8. According to calibration test and field test, this sensor, which features low cost, good operability, and high integration, is qualified for precise agricultural irrigation with stable performance and high accuracy.

## 1. Introduction

As a key factor influencing crop growth and yield, soil moisture content is an important basis for the management of agricultural production [1]. To realize intelligent farmland irrigation, it is important to measure soil profile moisture content accurately in real-time, to understand spatial water utilization, and to provide a basis for selection of time and threshold of optimum irrigation and development of precision irrigation [2,3]. Currently, the burial depth of soil moisture sensing sensor for moisture measurement in the soil vertical profile has not been systematically investigated. Most soil moisture sensors are needle-type with a typical needle length of 3–20 cm [4], which makes them only suitable for topsoil moisture content measurement rather than deep soil moisture measurement [5,6]. For the purpose of soil vertical profile moisture measurement, multiple soil moisture sensors are usually buried at different soil depths. This method not only requires substantial investment of time and effort, but the in-situ soil measurement environment is also vulnerable to disturbance during sensor arrangement, making it difficult to ensure consistency between the performance of multiple sensors [7,8,9,10].

To solve the difficulties of soil profile moisture measurement with the needle-type sensor, non-contact soil profile moisture measurement methods have been investigated both in China and abroad [11,12,13,14,15,16,17,18]. Yuan et al. [19] designed an equally spaced three-depth soil profile moisture sensor based on the impedance method. With a radial measurement radius of 3–4.1 cm and equal spacing of 20 cm, this sensor would result in information redundancy in practice. A tubular dielectric moisture sensor measurement system similar to EnviroSCAN was designed by Zhou et al. [20]; however, the system requires the use of a motor for horizontal migration of the single-point sensor at a single soil depth, making it inapplicable to automatic measurement of soil moisture content at different depths. Similar to Diviner2000 (Sentek, Australia), the manual single-point soil profile moisture sensor developed by Fu et al. [21] requires manual lifting as it is not able to achieve automatic moisture measurement in the vertical profile. Currently, the products with the widest applications in this field are the EnviroSCAN soil moisture profiling system [22,23] and the Diviner2000 soil moisture profiler [24]; however, both of these are expensive.

In general, current soil profile moisture measurement requires large numbers of soil moisture sensors at different soil layers, whereas data redundancy, a cumbersome process, and high cost make existing equal-spacing soil profile moisture sensors incapable. Given the lack of detailed information relating to soil profile moisture, intelligent irrigation is in great need of a low-cost and high-efficiency soil profile moisture sensing system to reduce the cost of soil moisture measurement and enhance irrigation efficiency [25,26].

Non-destructive sensing systems for soil moisture include time domain reflectometry (TDR), frequency domain reflectometry (FDR), and high-frequency capacitance (HFC). The sensing accuracy of TDR can only be ensured when the rise time of the drive signal is shorter than 200 ps. Such technical restriction in rise time makes it difficult to apply the TDR method to soil profile moisture monitoring [27,28,29]. Complex secondary calibration of FDR is required before use since it is subject to the effects of various factors, such as soil type, conductivity, and volumetric weight, when measuring frequency is low [17,30]. With simpler operation and higher efficiency than FDR, which requires frequency domain reflection, HFC is considered the best substitute for TDR and is able to satisfy the demands for real-time online measurement since it is not affected by soil texture in the HFC field [31,32].

Previous studies on soil profile moisture sensors have revealed various problems, such as high cost, high data redundancy, and complex operation [7,33,34,35,36,37,38,39]. In this study, a soil profile moisture sensor was designed following the principle of HFC, based on the research achievements on an efficient soil profile moisture sensing method developed by the National Engineering and Technology Center for Information Agriculture, Nanjing Agricultural University. With this device, not only could volumetric moisture content of the 20, 30, and 50 cm soil layers be detected, but online inversion of moisture for 0–100 cm soil depth could also be conducted. 

## 2. Design of the Soil Profile Moisture Sensor

### 2.1. The Sensing Principle

In the HFC method, soil moisture is obtained by measuring the equivalent capacitance of the soil mix (water, air, and soil particles) that serves as a medium between metal electrodes at a high frequency. Soil can be classified into sandy, clay, and loam soils by particle size. Dielectric constant of soil can vary significantly with different particle sizes and air spaces. However, HFC is immune to soil texture [31]. According to Hilhorst [40], dielectric constants of three types of soils were almost the same when the frequency of external electric field was 100–500 MHz and the influence of soil texture on the dielectric constant was negligible. The capacitance of the capacitor formed by the metal electrodes and soil is almost entirely determined by the soil moisture content since the dielectric constants of solid matters and gases are far smaller than that of water in soil. Therefore, soil moisture content can be estimated from the measured capacitance.

**Figure 1:**
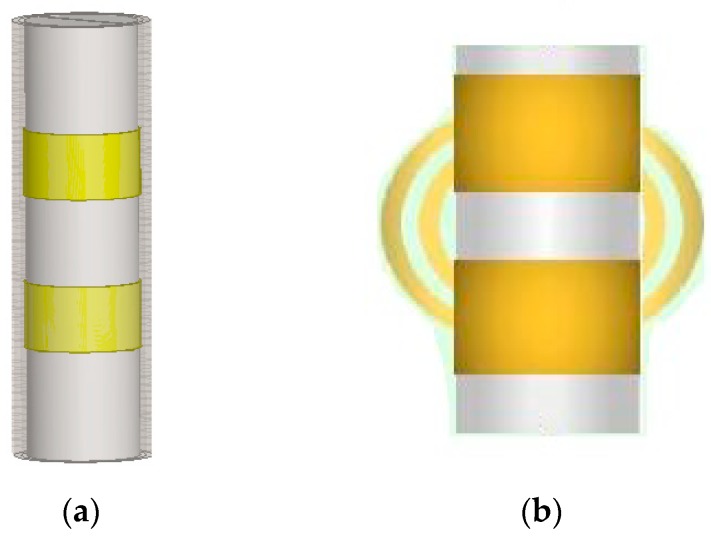
Structural diagram of the annular electrode: (**a**) The annular electrode; (**b**) The radiation electric field

In order to prevent the sensor from being affected by the soil corrosion, the annular electrode of the sensor was constructed as shown in Figure 1a. Two identical metal cylindrical rings were sheathed on the insulating sleeve, and the outer layer of the sensor was covered with an insulating tube layer to obstruct the soil corrosion. 

**Figure 2:**
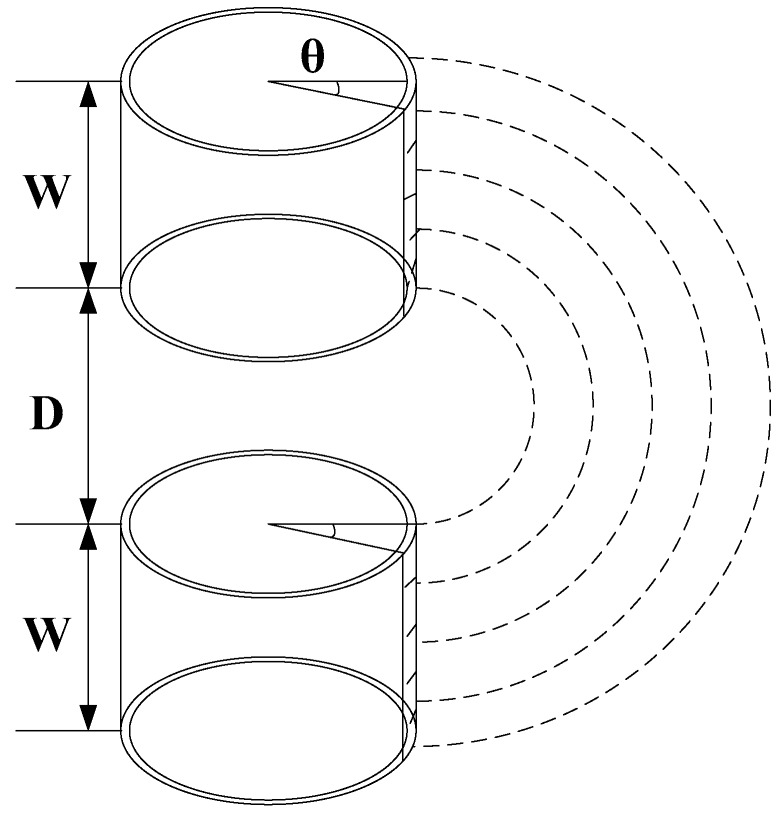
The physical structure of the annular electrode.

As shown in Figure 1b, when the two metal annular electrodes were connected to the circuit and radiating the electric field around the periphery, the measured soil and the two electrodes covered by the radiation electric field constituted an equivalent capacitor together. The physical structure of the annular electrode was shown in Figure 2.

**Figure 3:**
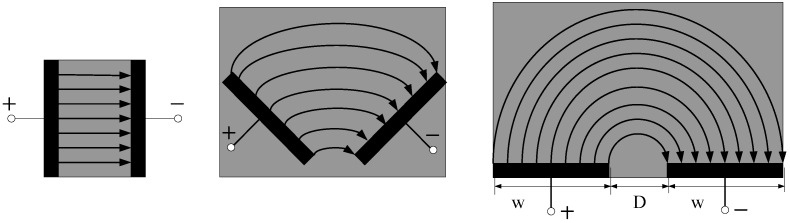
Diagram of parallel plate capacitance expanding to fringing field capacitance.

In Figure 2, *D* was the axial distance between two electrodes. Because the probe is based on the fringe field effect to sense the capacitive resistance of the water-containing soil, which is very similar to the working principle of the planar capacitance sensor. Therefore, the central angle *θ* was 1° in this paper, and the annular electrode was divided into 360 parts, and *θ* for each upper and lower annular electrode is 1°. The corresponding longitudinal section constituted a micro-quantized planar capacitance, as shown in Figure 3. 

**Figure 4:**
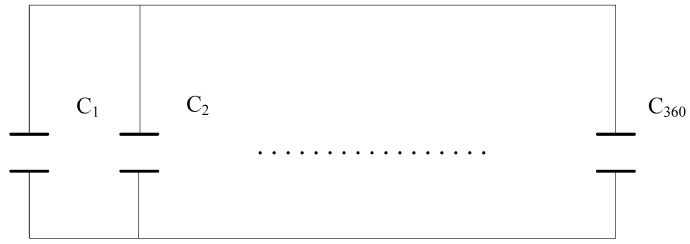
The annular probes sensing the equivalent capacitive reactance represented by soil.

As shown in Figure 3, the equivalent capacitance can be considered as parallel plate capacitors formed in parallel by an infinite number of micro-dimension fringing field capacitances. After the annular probe is micro-divided, the entire probe sensing the capacitive resistance represented by the soil based on the high-frequency fringing electric field effect can be equivalent to 360 micro capacitors in parallel, as shown in Figure 4.

The capacitance of the probe’s micro-quantized planar capacitance shown in Figure 4 was calculated using an approximate analytical algorithm, based on the relevant research of the fringing field capacitor [41]. In the solution-like algorithm, the unit integration method was taken as an example. Generally, the power line between two electrodes was considered as an arc curve or an elliptic curve [42]. The approximated solution of a single micro-quantized capacitance of an annular electrode was shown in equation (1):

(1) $$C_{i}=\frac{k_{0}R\theta_{i}(\varepsilon_{r}+\varepsilon_{0})}{\pi }\ln(1+\frac{2W}{D})$$

Among them,

$C_{i}$ —— Single micro-quantized capacitance
*k*_0_ —— Correction constant
*R* —— Radius of the annular electrode
$\varepsilon_{r}$ —— Dielectric constant of water-containing soil
$\varepsilon_{0}$ —— Composite dielectric constant of other radiation-field media
$\theta_{i}$ —— The central angle of the annular electrode corresponding to the micro-quantized fringing field, which is made constant by 1° (*i* = 1, 2, 3 ... 360)
*W* —— Width of the annular electrode
*D* —— Spacing of annular electrodes

In the case of non-changed structural parameters (the annular electrode width *W*, spacing *D* and radius *R*), the single micro-quantized capacitance value $C_{i}$ is related to the dielectric constant ($\varepsilon_{r}+\varepsilon_{0}$), so the dielectric constant of the measured soil can be converted to measure the size of the capacitance represented by the electric field.

**Figure 5:**
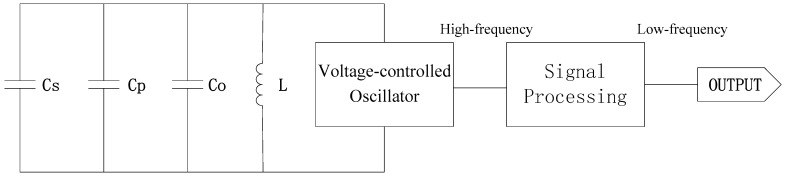
Equivalent circuit of the high-frequency capacitance method.

An easy way to measure capacitance is to place the equivalent capacitance characterized by the electrodes and soil into an LC oscillating circuit and measure the final circuit frequency. The equivalent circuit is shown in Figure 5. The voltage-controlled oscillator generates a high-frequency electromagnetic fringe field effect by means of fixed resonant capacitance and an inductor. The capacity of soil’s equivalent capacitance that is detected by the sensor’s probe is associated with the stray capacitance of surrounding soil and the probe itself [41]. Formed by this composite structure, this probe is equivalent to an equivalent capacitance with a stray capacitance. In order to allow the microprocessor to directly measure the output frequency, it is necessary to input the high-frequency signal into the signal processing frequency divider circuit, so that the signal is converted into a low-frequency signal output.

Equation (3) indicates that the frequency generated by a voltage-controlled oscillator is subject to the nominal values of capacitance and inductors in an LC resonance circuit. The dielectric constant of soil under test varies as the soil moisture content changes, which results in changes of its equivalent capacitance and circuit’s output frequency.

(2) $$f=\frac{1}{2\pi \sqrt{L(Co+Cp+Cs)}}$$

where *f* is the frequency; *Co*, *Cs*, and *Cp* are the fixed resonant capacitance, equivalent capacitance, and stray capacitance in the circuit, respectively; and *L* is the inductor. The dielectric constant soil characterized varies when soil moisture content changes, followed by the equivalent capacitance the probe detected, output frequency of high-frequency oscillator, and the DC voltage signals sent by the sensor after signal conditioning. Thus, the soil moisture content can be inverted based on the changing DC voltage signals.

### 2.2. Structural Design of the Sensor

**Figure 6:**
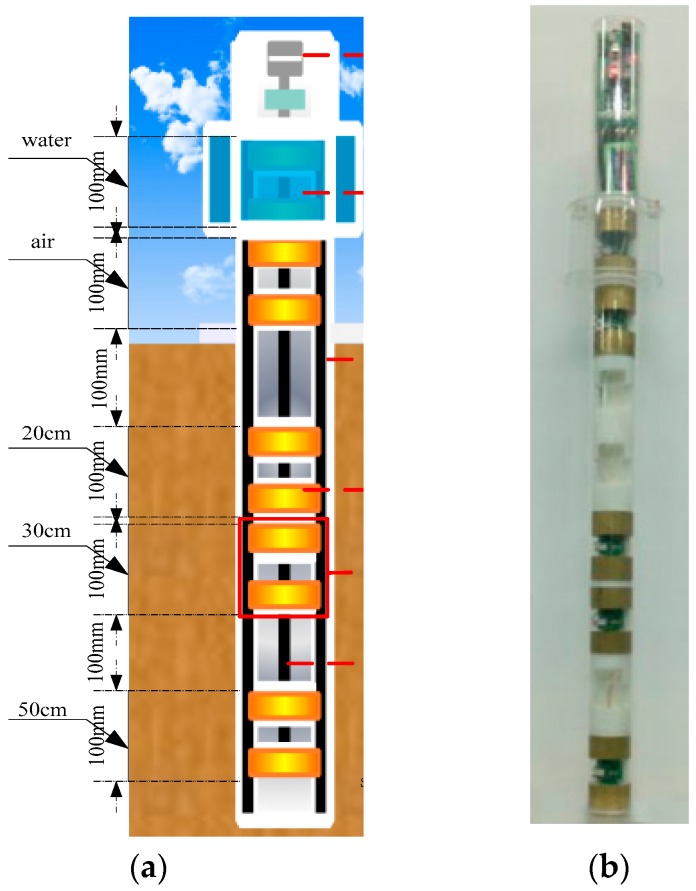
(**a**) Composition diagram and (**b**) physical product of the soil profile moisture sensor; ① Data processor; ② Water-layer capacitor; ③ Outer wall of the sensor; ④ Annular electrode; ⑤ Sensing probe; ⑥ Insulation sleeve.

As shown in Figure 6a, the sensor consisted of five sensing probes, a data processor, and accessory components. The five groups of sensing probes were capacitors comprising two annular electrodes. These five probes were designed to measure the water layer, air layer, and at soil depths of 20, 30, and 50 cm. 20, 30 and 50 cm represents the depth position of the sensor-sensitive probe in the soil, respectively. And the length of each layer is 100 mm. Capacitors for the air and water layers were able to dynamically calibrate each sensor.

The sensor was sealed using acrylic organic glasses. The five sensing probes were fixed to the insulation sleeve. The sensing probes and the data processor were connected to the power supply by shielded wires. At the bottom of the data processor, there were multiple sets of signal terminals used for signal frequencies outputted by various sensing probes. Signal interference and overpower during simultaneous function of multiple groups of sensing probes were avoided by time-sharing of the power supply. In order to avoid interference among electric fields and power overloading problems between adjacent sensor probes when the soil moisture content information is measured at all levels at the same time, five groups of sensor sensitive probes are operated by time-sharing control when the sensor is working. The solid-state relays (optical coupling isolation relay) of type AQY212S are used. The control port of the microprocessor emits high and low levels to drive the size of the output current, and large or small currents are fed into the front end of the solid state relay to control the connection and disconnection between two SWITCH ports at the rear end, respectively. The typical value of the relay turn-on time is 0.65 ms, and the closing time is only 0.08 ms.

### 2.3. Sensing Probe Design 

#### 2.3.1. Simulation Model of the Sensing Probe’s Annular Electrode

The electric field distribution around the annular electrode was determined by the electrode’s structural characteristics, which further affected the sensitivity of capacitance detection and range of detection. According to Equation (1), the size of the equivalent capacitance formed by the sensor probe adopting the annular electrode structure and the soil is affected not only by the vacuum dielectric constant and the relative dielectric constant, but also by the capacitance structural parameters (the annular electrode width *W*, the spacing *D* and radius *R*). Therefore, simulation of the annular electrode’s different geometric sizes was required. The geometric sizes of the annular electrodes of the sensing probes were identified based on the distribution of electric field around them. Electrode width and spacing are denoted as *W* and *D*.

**Figure 7:**
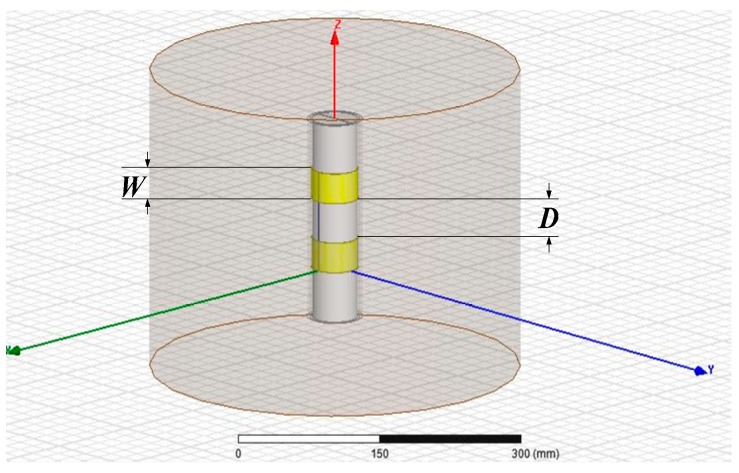
Simulation model of the annular electrode structure.

A high-frequency structure simulator (HFSS) from ANSYS Inc was used to simulate the radiation intensity of electric field generated by the annular electrodes and thus determine the optimal structure of the annular electrodes. A simulation model of the annular electrode structure was built. As shown in Figure 7, the external cylinder was soil space and the internal yellow ring was a copper ring whose inner and outer diameters were uniformly set to 48 mm and 51 mm, respectively. Suppose the boundary of electric field generated by the annular electrodes was ideal; the columnar soil space that was 40 cm in diameter and 30 cm in height was the boundary condition of radiation; the dielectric constants of the packing medium (soil volumetric moisture content was 34%) [41], the acrylic installation pipe and the shaft in the pipe, and air in the pipe were 21, 3.5, and 1, respectively; the solving frequency was 150 MHz, lumped port stimulation; *W* was set to 10, 20, and 30 mm; copper ring spacing was set to 10, 20, 30, 40, 50, and 60 mm.

#### 2.3.2. Simulation of the Sensing Probe’s Annular Electrode

The electric field distribution and shape obtained by the annular electrode simulation with different structure parameters varied. The effective lateral radiation range was determined by the diameter of the annular electrode. The effective radial radiation range and contiguity of the electric field were determined by the spacing between two annular electrodes. The results of the annular electrode simulation with different structure parameters are shown in Table 1. According to Table 1, when copper ring width (*W*) was 10 mm, the largest radiation range was observed when copper ring spacing (*D*) was 60 mm and axial and radial radiations were 100.00 mm and 80.00 mm, respectively. When *W* was 20 mm, the largest axial radiation range was observed when *D* was 40 mm and axial and radial radiations were 106.00 mm and 80.00 mm, respectively. The largest radial radiation range was observed when *D* was 60 mm and axial and radial radiations were 98.00 mm and 100.00 mm, respectively. When *W* was 30 mm, the largest axial radiation range was observed when *D* was 40 mm and axial and radial radiations were 94.50 mm and 100.00 mm, respectively; the largest radial radiation range was observed when *D* was 60 mm and axial and radial radiations were 90.00 mm and 120.00 mm, respectively.

**Figure 8:**
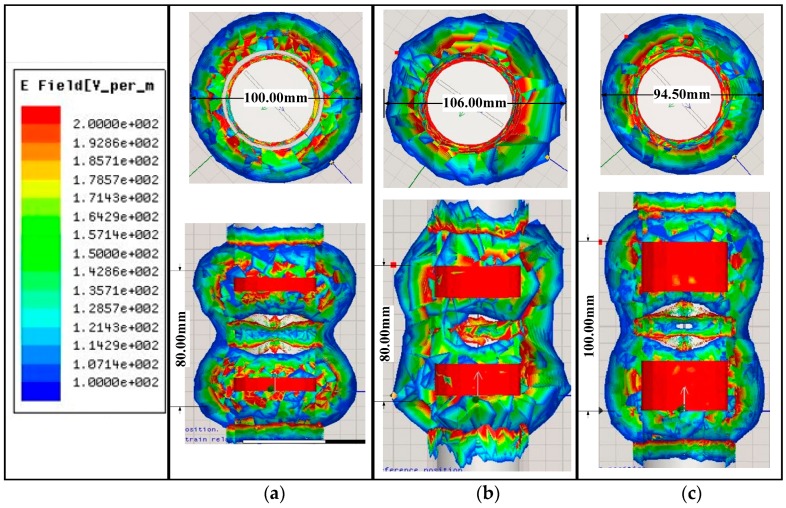
Comparison between the three combinations that have the largest axial radiation distance (**a**) *W* = 10 mm and *D* = 60 mm, (**b**) *W* = 20 mm and *D* = 40 mm, and (**c**) *W* = 30 mm and *D* = 40 mm.

A comparison of simulation results of three structure parameters with the largest distance of axial radiation is shown in Figure 8. When *W* was 10 mm and *D* was 60 mm, the radial radiation diameter was 100.00 mm with uniform distribution of field intensity, but the range of radial plane radiation was only 80.00 mm. When *W* was 20 mm and *D* was 40 mm, axial and radial field intensity was not uniformly distributed, the surrounding electric field was not compact, axial radiation diameter was as high as 106.00 mm, and the range of radial radiation was 80.00 mm. When *W* was 30 mm and *D* was 40 mm, the axial and radial field intensity was uniformly distributed; the axial radiation diameter was 94.50 mm, and the range of radial radiation was 100.00 mm. All things considered, the spacing of sensitive soil layer-sensing for the sensor was set to 100 mm, as well as the radiation range and continuity of the electric field, *W* = 30 mm and *D* = 40 mm were taken as the optimal structure of the sensor probe’s annular electrode.

### 2.4. Circuit Design

**Figure 9:**
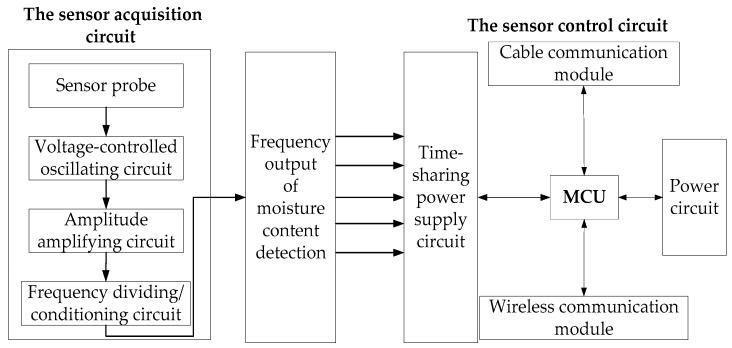
Design principles of the circuit of the soil profile moisture sensor.

The design principles of the circuit of the soil profile moisture sensor are shown in Figure 9. It functionally consisted of two parts, the sensor acquisition circuit, and the control circuit. The acquisition circuit comprises annular metal electrodes, voltage-controlled oscillating circuit, amplitude amplifying circuit, and frequency dividing/conditioning circuit, and it is used to monitor soil moisture content. The sensor control circuit comprises an ATmega328p 8-bit microcontroller (MCU), the power circuit, the 485 cable communication and transmission module, NRF 24L01 wireless 2.4 G communication and transmission module, and time-sharing power supply circuit and power circuit. The sensor was powered by a 7.4 V lithium battery and was accessed to the solar module.

**Figure 10:**
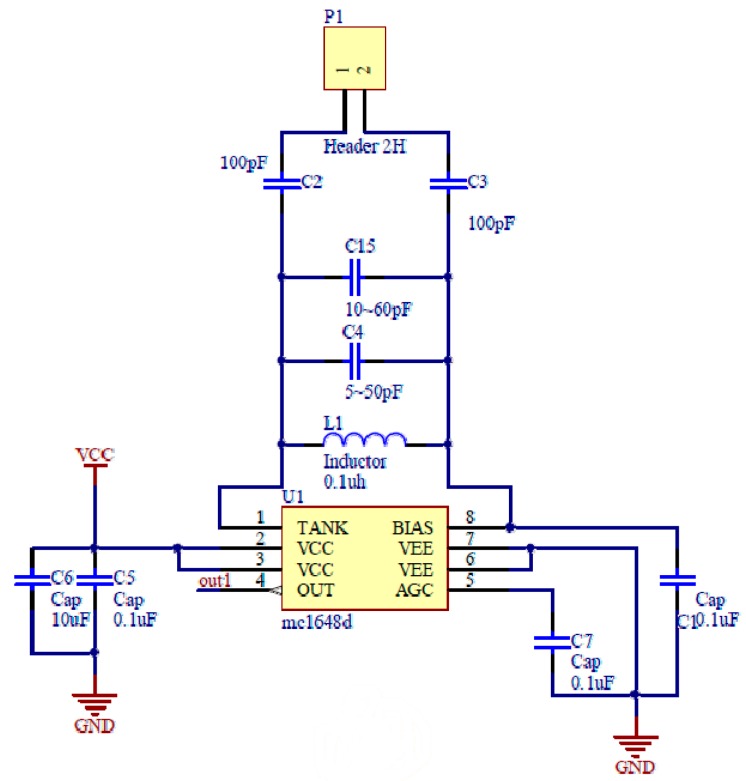
Schematic diagram of the LC resonance circuit.

Among them, the voltage-controlled oscillating circuit is different from the voltage-controlled oscillation mode application circuit commonly used in the VCO chip. An LC resonant mode application circuit of the voltage-controlled oscillation chip is used to place the equivalent capacitance formed by the annular electrode and soil in the LC oscillation circuit, so as to generate a variable high-frequency sinusoidal signal. The voltage-controlled oscillation chip uses the MC1648d from Freescale Company (currently NXP). The principle of voltage-controlled oscillator and LC resonance circuit is shown in Figure 10. An external LC and annular electrodes were arranged in parallel between Pin 1 (Tank) and Pin 8 (Bias) on the chip to make up the resonance tank, using the high-frequency oscillating circuit that outputs the oscillation frequency with a variable equivalent capacitance. As shown in Figure 10, the inductor L1 is encapsulated by the patch 0805 with the size of 0.1 μH order of magnitude. Both C2 and C3 are 100 pF capacitors encapsulated by the patch 0805, the connecting terminal P1 is used to connect two brass annular electrodes, the three of C2, C3, and P1 constitute the capacitance compensation circuit of the soil equivalent capacitance to avoid the input multivalued problem. Capacitor C4 and C15 use variable capacitors in the order of several tens of pF as resonant capacitors by adjusting the size of the variable capacitor. On the one hand, the sensitivity and the range of the soil equivalent capacitance to the output frequency are adjusted. On the other hand, the inherent error caused by the structure and component accuracy of multiple sensor circuits are also adjusted, so as to ensure the consistency of the output of each sensor. C1, C5, C6, and C7 are all patch capacitors encapsulated by 0805, where C6 is different from C1, C5, and C7 in order of magnitude, C5 and C6 are used as decoupling capacitors to filter out high and low-frequency noise interference from the power supply and the ground plane, respectively. C1 and C7 are used as filter capacitors to filter out the interference of parasitic impedance to the oscillating loop in high-frequency circuits.

Signal processing primarily refers to the frequency division, conditioning, and shaping of frequency signals, i.e., conducting frequency division of original frequency signals to allow the MCU to measure them directly. The change of soil moisture content leads to the variability of the equivalent capacitance within a certain range. The output high frequency actually measured by the voltage-controlled oscillating circuit is between 100 MHz and 150 MHz. To enable the measured output frequency can be read by the microprocessor, it is necessary to input high-frequency signals into the signal processing frequency divider circuit and also input transfer high-frequency signals into low-frequency signals. In this study, the original frequency signals were divided by 64 using the dual-mode pre-scaler and then divided by 256 using a SN74HC393D digital chip, and the result was the measurement frequency. To enable the back-end processor to measure this frequency signal directly, MB504 and SN74HC393D typical frequency dividing circuits were used in this study [13]. SN74HC393D’s digital chip is a dual four bit binary counter produced by TI. The maximum count range is 256. When the 256 signal pulses are input, the output will be flipped and the 256 frequency of the input frequency will be realized.

### 2.5. Software Design

**Figure 11:**
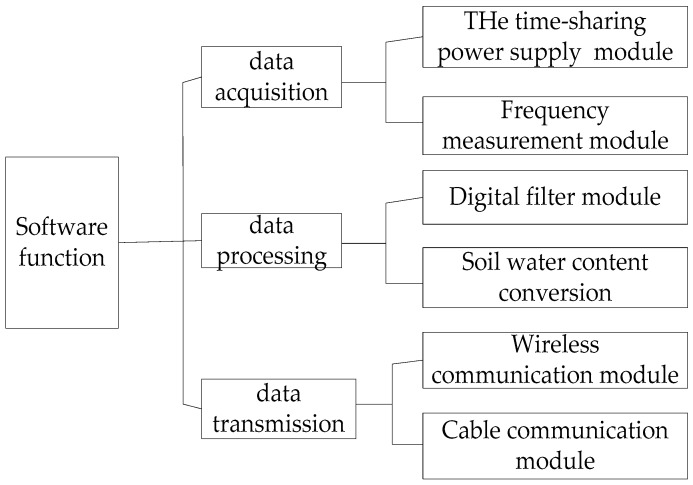
Block diagram of the software functions.

According to system requirements, the software realized the acquisition of soil profile moisture data and data processing and transmission. The major function of data acquisition was to acquire the output frequencies of sensing probes on five groups of sensors, i.e., calling the time-sharing power supply program to switch on the power of the acquisition circuit corresponding to the current loop variable and select to open the channel for the input of frequency signals of this acquisition circuit to complete data acquisition. Data processing was designed to call the digital filtering module to remove invalid measured data among multiple frequency signals, obtain the final frequency value, and convert the measured frequency value into the volumetric moisture content of soil sample under test using the soil moisture content conversion subprogram. The detailed procedure is presented as followed. Firstly, to make a macro definition on the sample number with a constant N fixed at 101. Secondly, cycle sampling N times with calling the measurement function every 1 ms, and assign the output value to an array space with a space size of N. Thirdly, all the values in the space are sorted by subscripts from largest to smallest. Finally, the program is ejected and median values of the array space are returned as the filter output result. Data transmission was intended to “pack” and “split” soil profile moisture after it was acquired and processed, split variables based on data size to ensure smooth data transmission, and guarantee that the data at the receiving end were merged and restored to obtain the original variable values. A software function block diagram is shown in Figure 11.

## 3. Performance Test Design, Data Analysis Methods, and Results

### 3.1. Performance Tests 

#### 3.1.1. Laboratory Test

**Figure 12:**
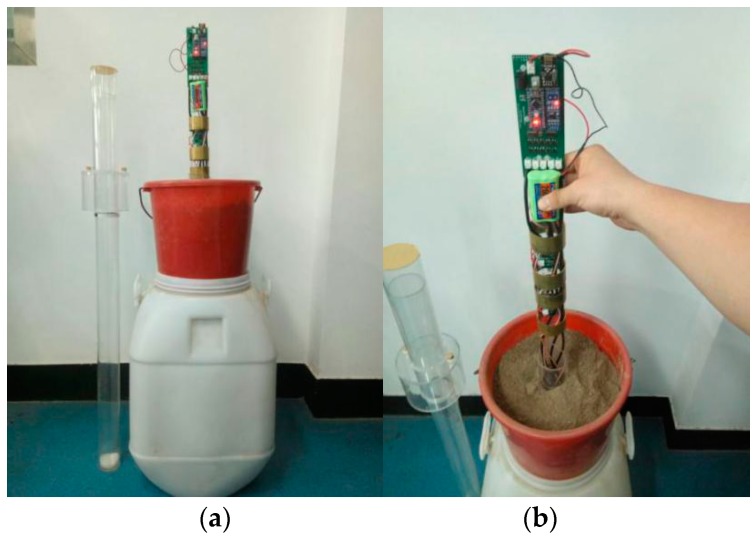
Photographs of the experimental set up. (**a**)Test equipment, (**b**) Operation process.

A concentric hole the size of the sensor’s outer-wall diameter was excavated on the bottom of a plastic bucket with an opening diameter, bottom diameter, height, and volume of 310 mm, 210 mm, 280 mm, and 12 L, respectively. Next, a 350 mm-long insulation tubular column was inserted into the hole and fixed and sealed with hot-melt adhesive. The sensor was then inserted into the soil samples where the insulated outer-wall was fixed. The shaft was manually moved up and down such that sensor probes at different levels could be immersed in the soil. A testing image is shown in Figure 12.

(1) Calibration of Soil Volumetric Moisture Content

Loam soil samples were collected in Rugao, Jiangsu Province, China, with a volumetric weight of 1.12 g/cm^3^. In a laboratory with a constant temperature in the range 15–25 °C, the samples were divided into nine density gradients with the volumetric moisture content from low to high until nearly saturated (i.e., 0.00, 0.07, 0.13, 0.20, 0.26, 0.31, 0.37, 0.47, and 0.59; unit: m^3^/m^3^) and then stored in a sealed barrel for 48 h. The soil sample of every gradient was measured 10 times with one of the soil profile moisture sensor’s probes (30 cm). The soil prepared to be tested is weighed via the electronic balance scale with the accuracy of 0.01 g. Then, the soil after being weighed will be dried out via the electrothermal blowing dry box before the dry soil being weighted again. With the oven-drying method, the actual water content of the volume of the tested soil is calculated. The functional relationship between output frequency of acquisition circuit at the sensor’s measuring end and actual soil volumetric moisture content was determined. In the same environment, soil samples of five other gradients (0.11, 0.25, 0.32, 0.46, and 0.51; unit: m^3^/m^3^) were measured with the same method. The data were substituted into the functional relationship to test the accuracy of calibration of the soil volumetric moisture content.

(2) Consistency Test

Test materials and environment that were identical to those of the soil volumetric moisture monitoring model were used to eliminate individual differences. The five probes were inserted into the barrel to measure each kind of moisture treatment. Actual soil volumetric moisture content was obtained with the oven-drying method.

(3) Verification Test with Different Soil Types

**Figure 13:**
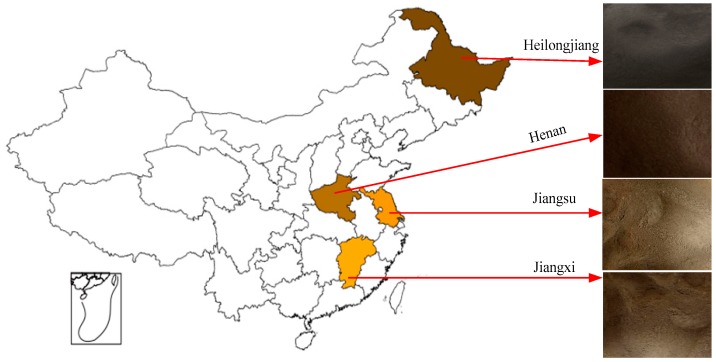
Sampling regions and types of soil samples.

To verify whether the proposed sensor was widely adaptable to different soil types, the soils used for test were collected from China’s four major agricultural provinces, namely yellow brown soil from Jiangsu Province, sandy loam from Jiangxi Province, clay loam from Heilongjiang Province, and sandy loam from Henan Province. Concrete distribution of soils is shown in Figure 13. Soils underwent pretreatment, including weed removal, air drying, grinding, and sieving, before they were placed in the sealed barrel for measurement.

The soil samples collected from Jiangxi Province, Henan Province, and Heilongjiang Province were prepared to different soil volumetric moisture contents using the same method as above. Density gradients of soil samples were variable among samples since volumetric weight and soil moisture content of soil samples collected from one region was different to those of soil samples collected from another region. The prepared moisture contents of soil samples from Jiangxi Province were 0.00, 0.10, 0.14, 0.21, 0.28, 0.31, 0.43, and 0.48 m^3^/m^3^; the prepared moisture contents of soil samples from Henan Province were 0.00, 0.10, 0.19, 0.26, 0.39, and 0.52 m^3^/m^3^; and the prepared moisture contents of soil samples from Heilongjiang Province were 0.00, 0.10, 0.17, 0.27, 0.32, 0.44, and 0.56 m^3^/m^3^. The sensor was then inserted into the plastic tubular column in the center of measuring barrel to measure each soil sample ten times. When measurements were completed, the actual volumetric moisture content of soil samples under test was obtained using the oven-drying method.

#### 3.1.2. Field Test

**Figure 14:**
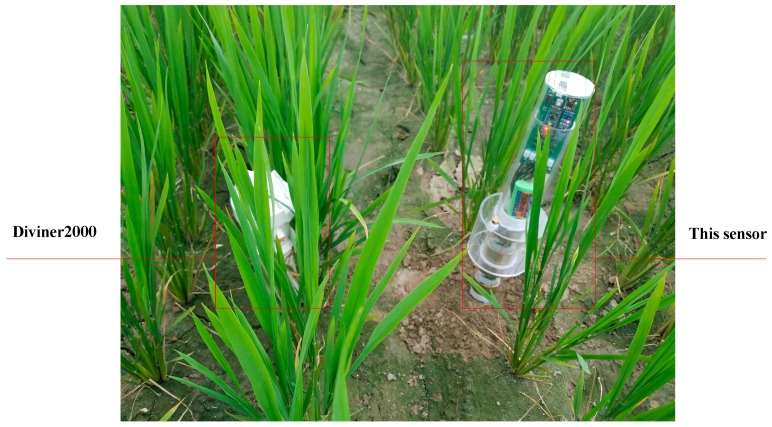
Photograph of the field test design.

The test was carried out in the Rugao Demonstration Base of National Engineering and Technology Center for Information Agriculture, Nanjing Agricultural University from July to October 2017. The soil type of the test site was silty loam. Field test setup and comparison between tests are shown in Figure 14. With a measurement accuracy of 1%, the Diviner2000 portable soil moisture monitoring system was used for comparison. A 120 cm-long monitoring annular pipe with a 15 cm aboveground part was and 105 cm underground part was installed to Diviner2000 and kept sealed in the test plot before test, so that the system was able to measure the soil volumetric moisture content of vertical soil profile at the depths of 10, 20, 30, 40, 50, 60, 70, 80, 90, and 100 cm quickly and automatically. Similar to Diviner2000, a 100 cm-long monitoring annular pipe whose aboveground and underground parts were both 50 cm was installed to the proposed sensor in the test plot before test, to allow the rapid automatic measurement of the soil volumetric moisture content of vertical soil profile at the depths of 20, 30, and 50 cm.

Volumetric moisture contents of different soil layers were obtained during the field test. Then, linear regression analysis of measurement results was carried out. The volumetric moisture content of soil layers between 0–100 cm was calculated using the multiple regression equation developed based on sensitive soil layers. The feasibility of moisture content detection based on sensitive layers was verified from the quality of fit between the calculated and measured data.

#### 3.1.3. Data Analysis Method

(1) Drying method

Samples are classified via the soil volume moisture content volume when the water content of mass is calculated for the quantity of the samples. The conversion relation between two factors is shown as follows:

(3) $$\theta_{v}=\theta_{m}\times \rho_{b}$$

In the above equation, $\theta_{m}$ represents the mass water content of soil, $\theta_{v}$ represents the volume water content while $\rho_{b}$ represents the volume weight of the dry soil.

$\rho_{b}$ was calculated as the ratio between the mass of the dry soil and the volume of the dry soil with the unit of g/cm^3^, as followed.

(4) $$\rho_{b}=\frac{W_{s}}{V_{0}}$$

$V_{0}$ represents the total volume of the soil while $W_{s}$ represents the mass of the earth in the soil. The equation for calculating the mass water content of soil $\theta_{m}$ is shown as followed:

(5) $$\theta_{m}=\frac{W_{w}}{W_{s}}\times 100\%$$

In the equation of (5), $W_{w}$ represents the mass of water in the soil. The weighing method of oven drying is applied. The water content value of the mass of the soil is calculated before the actual volume water content of the soil samples is finally obtained from the equation (3).

(2) Automatic calibration calculation

The sensor measures the frequency of the soil under the surface of the solid phase medium, which is $X_{iSoil}$, the gas-phase medium exposed to the air on the surface of the earth of $X_{air}$ and the frequency of liquid-phase medium water wrapped in a hollow water column of $X_{water}$. The self-calibration formula for the three-phase $Y_{iwater}$ normalized frequency value in the soil at the depth *i* is:

(6) $$Y_{iwater}=\frac{X_{air}{-X}_{isoil}}{X_{air}-X_{water}}$$

Through the relationship between capacitance and dielectric constant $\varepsilon =C_{soil}/C_{air}$, the dielectric constant of the perceived soil can be calculated. $C_{air}$ is the capacitor when the medium is the air, $C_{soil}$ is the capacitor when the medium is soil. Based on the linear relationship between soil permittivity and soil moisture content: $\theta_{iwater}=a\sqrt{\varepsilon }+b$. Among them, *a* and *b* are constants and are closely related to the type of soil to be measured.

(3) Calibration

A soil volumetric moisture content monitoring model was built based on the calibration test. The root mean square error (RMSE) was used as the index of accuracy of the model:

(7) $$RMSE=\sqrt{\frac{\sum_{1}^{n}\left(\theta_{vi}-{\hat{\theta }}_{vi}\right)}{n}}$$

where $\theta_{vi}$ is the measured volumetric moisture content of the ith soil sample and ${\hat{\theta }}_{vi}$ is the volumetric moisture content obtained by the *i*th soil sample’s sensor.

(4) Consistency Test and Verification Test with Different Soil Types

The sensor’s output stability was tested using the relative error (*RE*) between the measured value and the actual value from the consistency test and verification test:

(8) $$RE=\frac{\left|\theta_{v}-{\hat{\theta }}_{v}\right|}{\theta_{v}}\times 100\%$$

where $\theta_{v}$ is the measured volumetric moisture content of the soil sample and ${\hat{\theta }}_{v}$ is the volumetric moisture content obtained from the soil sample sensor.

(5) Field Test Accuracy

The field test accuracy of the sensor was determined by comparing the proposed sensor and the Diviner2000 portable soil moisture monitoring system. The coefficient of determination (*R*^2^) was used to evaluate the accuracy:

(9) $$R^{2}=\frac{\sum_{i=1}^{n}\left(y_{ai}-{\bar{y}}_{a}\right){\left(y_{pi}-{\bar{y}}_{p}\right)}^{}}{\sqrt{\sum_{i=1}^{n}{\left(y_{ai}-{\bar{y}}_{a}\right)}^{2}{\left(y_{pi}-{\bar{y}}_{p}\right)}^{2}}}$$

where $y_{ai}$ and $y_{pi}$ are the measured and predicted moisture content of the whole soil profile at time *i*, respectively; ${\bar{y}}_{a}$ and ${\bar{y}}_{p}$ are the means of n measured and predicted values, respectively. The closer the $R^{2}$ to 1, the higher the correlation between the measured and predicted values.

The moisture content of the whole soil profile was predicted using the optimal combination when the volumetric moisture content measured in sensitive soil layers was the independent variable. Specifically, soil profile moisture content was verified by means of quantitative inversion. The goodness of fit between the predicted value and measured data was calculated using the multiple regression equation built based on sensitive soil layers, through which the feasibility of moisture content detection based on sensitive soil layers was verified. The volumetric moisture content of soil in deep sensitive layers served as a controlled variable *X_i_* to calculate the ternary linear regression equation of Y, the volumetric moisture content of the whole soil profile.

**Figure 15:**
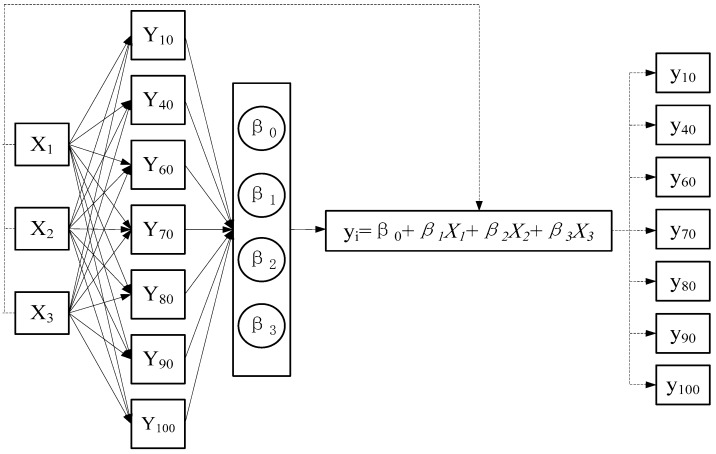
Process of multiple linear regression verification.

Suppose the linear regression model between random *y* and general variables $X_{1},X_{2},\ldots ,X_{k}$ was

(10) $$y=\beta_{0}+\beta_{1}X_{1}+\beta_{2}X_{2}+\beta_{k}X_{k}+\varepsilon$$

where $\beta_{1},\beta_{2},\ldots ,\beta_{k}$ are *k +* 1 unknown parameters; $\beta_{0}$ is regression constant; $\beta_{1},\beta_{2},\ldots ,\beta_{k}$ is regression coefficient; *y* is the explained variable; and $X_{1},X_{2},\ldots ,X_{k}$ are general variables that can be controlled precisely, known as explanatory variables. A performance comparison and verification model is shown in Figure 15.

As shown above, soil volumetric moisture content measured by Diviner2000 portable soil moisture monitoring system at the depths of 20, 30, and 50 cm were taken as controlled variables {X_1_, X_2_, X_3_}, which were then used to construct the ternary linear regression equation with {Y_10_, Y_40_, Y_60_, Y_70_, Y_80_, Y_90_, Y_100_} representing the soil volumetric moisture content measured at the depths of 10, 40, 50, 60, 70, 80, 90, and 100 cm. Soil volumetric moisture content measured by the proposed sensor at the depths of 20, 30, and 50 cm served as the input and the ternary linear regression equation as the inversion model to calculate the volumetric moisture content of the other soil layers at the depths of 10, 40, 50, 60, 70, 80, 90, and 100 cm, i.e., values of {y_10_, y_40_, y_60_, y_70_, y_80_, y_90_, y_100_}.

### 3.2. Results 

#### 3.2.1. Calibration of the Soil Volumetric Moisture Content Model

**Figure 16:**
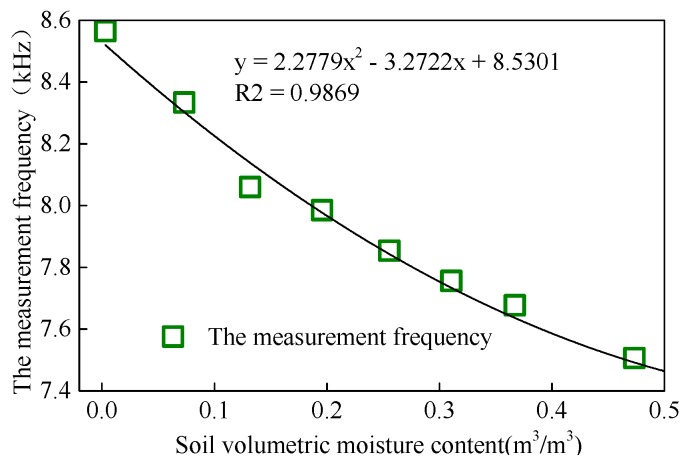
Calibration result of soil samples from Rugao (loam).

As shown in Figure 16, the measurement frequency of the sensor gradually declined as the volumetric moisture content of soil sample increased. Using the least squares method, the *R*^2^ between the two was found to be 0.9869, indicating that the frequency measured by the sensor was significantly correlated with the volumetric moisture content of the soil sample under test.

**Figure 17:**
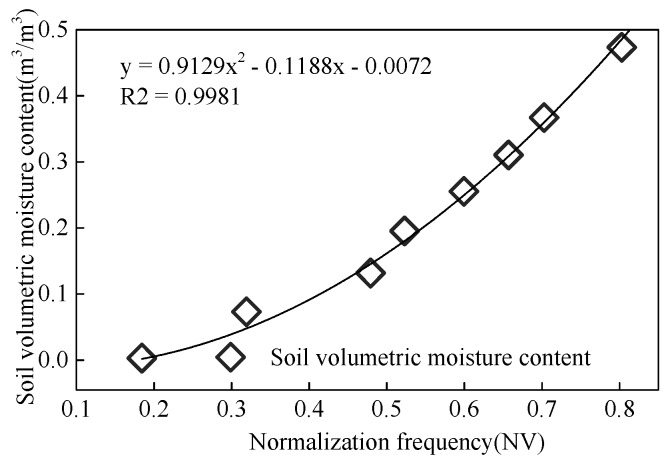
Normalized calibration result.

Due to the error exists between the actual value of the system frequency and the nominal value, it will cause the deviation between the testing frequency output from the sensor and actual frequency. To gain the conversion relationship between the actual testing frequency and the soil volume moisture content, the testing frequency is normalized with the establishment of function relationship between normalized results and the soil volume moisture content. As shown in Figure 17, the *R*^2^ of this relationship was 0.9981.

**Figure 18:**
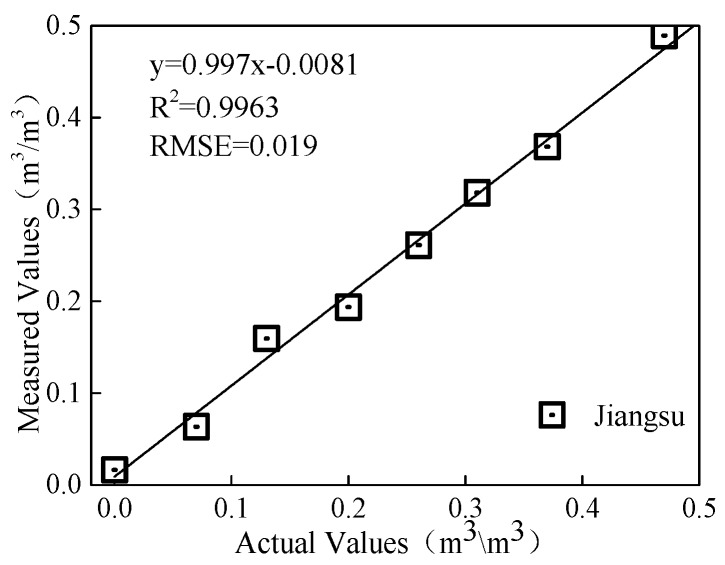
Verification result of soil volumetric moisture content monitoring model.

Soil volumetric moisture content monitoring model built based on the calibration test was verified. As shown in Figure 18, the *R*^2^ was 0.9991 and RMSE was 0.0186, indicating good accuracy of the proposed sensor. The objective of the nominal test is to determine the direct relationship between the collection circuit output frequency and the water content of actual soil value. The measuring algorithm of the sub-modules for converting soil water content in the software system is confirmed via the nominal test results. 

#### 3.2.2. Results of the Consistency Test

The results of consistency test are shown in Table 2. With the frequency measured by water-layer probe as the reference value, the relative errors between frequency values measured by the four other probes and reference value were calculated to be in the range 0–1.17%, indicating that the results measured by five probes showed favorable consistency.

**Figure 19:**
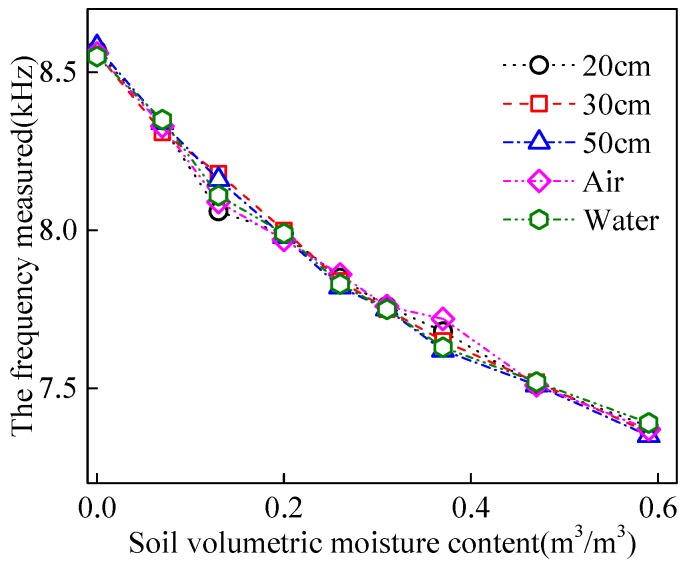
Fitting result between the values measured by five probes and soil volumetric moisture content.

Figure 19 shows the regression between measurement frequencies of five probes and the measured soil moisture contents. The fitting curves of five probes were overlapping in the figure, indicating good consistency between the sensor probes developed in this study.

#### 3.2.3. Verification of Different Soil Types

**Figure 20:**
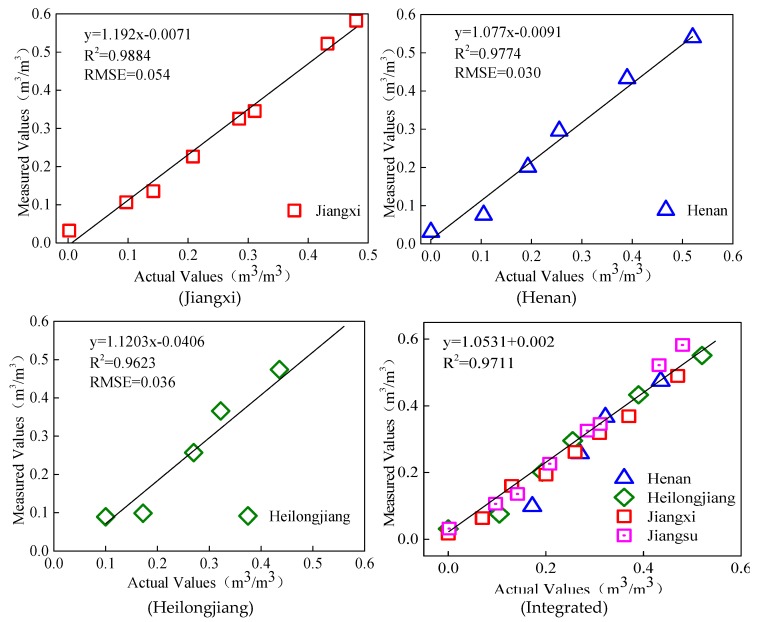
Integrated verification of volumetric moisture content of different types of soil samples.

Regression analysis between volumetric moisture content of different types of soil samples and soil moisture content measured by the sensor (Figure 20) indicated that the *R*^2^ and RMSE of soil from Jiangxi Province (sandy loam) were 0.9962 and 0.0535, respectively; those of soil from Henan Province (sandy loam) were 0.9774 and 0.0300, respectively; and those of soil from Heilongjiang Province (clay loam) were 0.9623 and 0.0364, respectively. As a whole, the *R*^2^ of linear regression between measured values and actual values in all four regions was 0.9644 and the RMSE was 0.0423. The *R*^2^ values between the soil moisture measured by the proposed sensor and actual soil moisture content were higher than 0.96 for all soil types. They were significantly correlated, with a small RMSE, suggesting that the proposed sensor was favorably adaptable to the measurement of volumetric moisture content of various soil types.

#### 3.2.4. Field Test Accuracy

**Figure 21:**
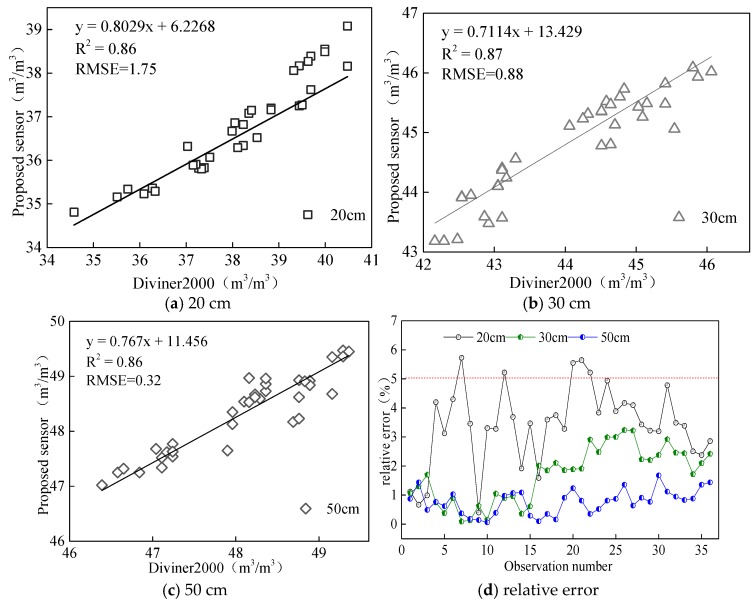
Verification of measuring performance at (**a**) 20 cm, (**b**) 30 cm, (**c**) 50 cm, and (**d**) relative error.

Comparison of measurement results between the proposed sensor and the Diviner2000 soil moisture monitoring system is shown in Figure 21. The *R*^2^ and RMSE between the value measured by the proposed sensor and soil volumetric moisture content from Diviner2000 were 0.86 and 1.75, respectively, at 20 cm soil depth; 0.87 and 0.87, respectively, at 30 cm soil depth; 0.86 and 0.32, respectively, at 40 cm soil depth; and 0.94 and 0.38, respectively, at 50 cm soil depth.

According to the relative errors between the results measured by the proposed sensor and Diviner2000 (Figure 21d), the proposed sensor’s maximum relative measurement error was 5.73% and it was smaller than 5% at for all soil depths except 20 cm. This indicates that the measurement accuracy of the proposed sensor was comparable with that of Diviner2000 and met the requirements for application.

Ternary linear regression of soil volumetric moisture content at depths of 10, 40, 50, 60, 70, 80, 90, and 100 cm were constructed with the soil volumetric moisture content measured at the depths of 20, 30, and 50 cm by Diviner2000 as the controlled variable. As shown in Table 3, the *R*^2^ of regression equations for predicted depths of 10 cm, 40 cm, and 60 cm are higher than 0.9, and the *R*^2^ of regression equations for other predicted depths are higher than 0.85. The highest *R*^2^ of 0.984 was found for regression equation for predicted depths of 40 cm layer in a loam soil.

**Figure 22:**
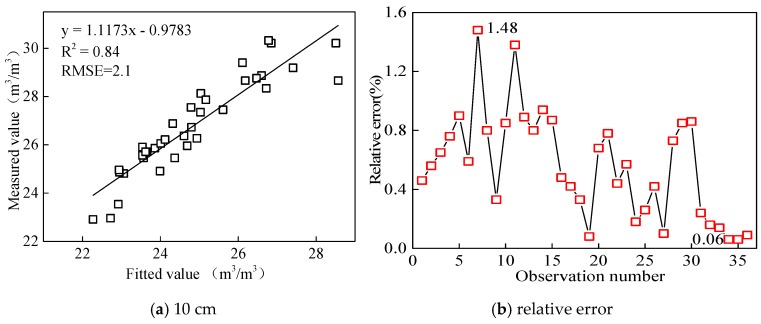
Correlation and relative error between fitted value and measured value at the depth of 10 cm.

The volumetric moisture content of 10 cm soil depth measured by Diviner2000 was taken as the measured value, and ternary regression was carried out with the soil volumetric moisture contents measured by the proposed sensor at the depths of 20, 30, and 50 cm (Figure 22). The *R*^2^ was 0.84, and the relative error between fitted values and measured value was 0.06–1.48% (<5%). 

**Figure 23:**
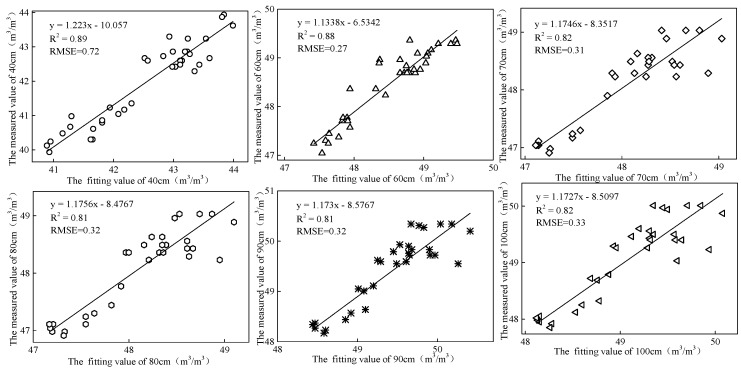
Correlation between fitted values and measured values at different depths.

Fitting of volumetric moisture content of the other soil layers was carried out according to the volumetric moisture content measured by the proposed sensor at the depths of 20, 30, and 50 cm. The correlation between the fitting values of 40, 60, 70, 80, 90, and 100 cm-deep soil layers and measured values (Figure 23) produced *R*^2^ values of more than 0.8, which indicated that the moisture content of the other soil layers could be calculated based on the moisture content of sensitive soil layers measured by the proposed sensor. The soil volumetric moisture content of soil layers between 0–100 cm was favorably inverted by the proposed sensor, providing a new approach to the development of an efficient soil moisture sensing system.

## 4. Discussion

Development of an accurate soil profile moisture sensor remains the priority for intelligent farmland irrigation because traditional needle-type soil moisture sensors are vulnerable to erosion due to direct contact with soil, which results in low measurement accuracy [4]. In addition, inadequate needle length also makes it difficult to conduct simple and quick moisture sensing content in deep soil. To this end, a non-contact measurement structure based on annular-electrode probes was proposed in this study. The axial radiation diameter and range of radial plane radiation of the sensor’s sensing probes were 94.5 mm and 100 mm, respectively. The measurement radius of this sensor was 2.5 times that of the equally spaced three-depth soil profile moisture sensor developed by Yuan et al. [19]. 

Unlike TDR [8], the HFC method developed in this study can be directly applied to soil profile moisture monitoring. A sensor probe control circuit that enabled moisture content at different soil depths by means of time-sharing power supply was designed to avoid signal interference and power overload. The AVR single-chip microcontroller served as the MCU. Functions such as measuring the output frequency of acquisition circuit were realized at the hardware level, which enables the sensor to meet practical measurement demands. 

According to the comparison between the proposed sensor and Diviner2000 portable soil moisture monitoring system, relative error between the values measured by both sensors at the depth of 20 cm was higher than those at the depths of 30 cm and 50 cm, which might be because the 20 cm soil layer is close to the soil surface and the temperature of the topsoil is higher than that of the deep soil from July to September when the test was conducted. The field test of sensor accuracy indicated that quantitative inversion of moisture content of the other soil layers at the depth of 0–100 cm could be realized based on the moisture content of three soil layers measured by the proposed sensor. The *R*^2^ of regression equation between measured values and fitted values was higher than 0.85, which demonstrated favorable inversion of the volumetric moisture content at 0–100 cm soil depth by the proposed sensor. 

The findings of this research provide a new direction for the development of efficient moisture sensing systems. However, the proposed sensor might not be accurate in arid land since the modeling data were collected from the rice field. Therefore, further modeling is still required to verify its inversion function under other environmental conditions. Because only the influence of moisture variations in different soil types on sensor output was the focus of this study, the influence of physicochemical properties (e.g., soil temperature, volumetric weight, and conductivity) on the sensor’s measurement should be considered in future research.

## 5. Conclusion

A soil profile moisture sensor was developed based on the principle of HFC in this study. The simulation of sensor probe structure, design software, and hardware systems, and tests on the proposed sensor were also conducted.

(1) Soil moisture measurement following the HFC principle was used to design a sensor that can meet the demands for soil profile moisture measurement. A sensor that is able to conduct real-time detection of volumetric moisture content in three sensitive soil layers was proposed. Double copper rings were used as the component of annular electrodes of the proposed sensor’s sensing probe. Different structure sizes and electrode spacing of annular electrodes were simulated using HFSS. The probe structure for an optimal detection range was determined by means of simulation analysis (*W* = 30 mm and *D* = 40 mm).
(2) The hardware circuit was designed using the high-frequency LC in parallel with a resonance circuit comprising the voltage-controlled oscillator and annular electrodes. Frequency division and conditioning of frequency signals were performed. A software system was designed to realize real-time detection of soil profile moisture content. The calibration test of soil volumetric moisture content model produced an *R*^2^ of 0.9663. According to the performance test, different sensor probes had good consistency, with absolute relative errors between 0% and 1.17%. The sensor shows favorable adaptability to different soil types. Fitting between actual volumetric moisture content and values measured by the proposed sensor showed an *R*^2^ and RMSE of 0.9644 and 0.0423, respectively.
(3) According to the results of the sensor accuracy test, the *R*^2^ values between results measured by the proposed sensor and Diviner2000 were above 0.85, with relative errors less than 5%. With the soil volumetric moisture content measured at the depths of 20, 30, and 50 cm measured by the proposed sensor as the controlled variable, the *R*^2^ values between the calculated and measured soil moisture values of the other soil layers were all larger than 0.80 and thus quantitative inversion of volumetric moisture content of the other soil layers at the depth of 0–100 cm was successful. In conclusion, the sensor designed in this study has shown a promising level of performance and can be applied to practical measurement of soil profile moisture content at different depths.
(4) The test and the inversion of the water content in the soil profile are realized well from the sensing methods and the sensor developed here. However, current tested data are derived only from the soil in four different areas. Meanwhile, only the samples from the rice fields in plain areas are taken based on the analysis results of the sensitive soil layers. Before applying our sensor in the dry farms and other hilly areas on a large scale, compatibility tests should be carried out to obtain accurate and stable results from the sensor.

## Figures and Tables

**Table 1:** Simulation result of annular electrodes with different structure parameters (unit of radiation distance: mm).

Width (*W*)	Direction	Copper Ring Spacing (*D*)					
		10 mm	20 mm	30 mm	40 mm	50 mm	60 mm
10 mm	Axial	82.00	90.00	91.00	94.50	100.00	100.00
	Radial	30.00	40.00	50.00	60.00	70.00	80.00
20 mm	Axial	84.00	95.00	95.20	106.00	98.00	98.00
	Radial	50.00	60.00	70.00	80.00	90.00	100.00
30 mm	Axial	85.00	92.00	94.00	94.50	92.50	90.00
	Radial	70.00	80.00	90.00	100.00	110.00	120.00

**Table 2:** Measurement results and relative error (RE) from the five probes.

Volumetric Moisture Content (m^3^/m^3^)	50 cm (kHz)	30 cm (kHz)	20 cm (kHz)	Air Layer (kHz)	Water Layer (kHz)	50 cm RE (%)	30 cm RE (%)	20 cm RE (%)	Air layer RE (%)
0.00	8.57	8.56	8.58	8.56	8.55	0.23	0.12	0.35	0.12
0.07	8.33	8.31	8.34	8.33	8.35	0.24	0.48	0.12	0.24
0.13	8.06	8.18	8.16	8.09	8.11	0.62	0.86	0.62	0.25
0.20	7.99	8.00	7.98	7.97	7.99	0.00	−0.13	0.13	0.25
0.26	7.85	7.84	7.82	7.86	7.83	0.26	−0.13	0.13	0.38
0.31	7.76	7.75	7.75	7.76	7.75	0.13	0.00	0.00	0.13
0.37	7.68	7.65	7.62	7.72	7.63	0.66	0.26	0.13	1.18
0.47	7.51	7.52	7.51	7.51	7.52	0.13	0.00	0.13	0.13
0.59	7.38	7.36	7.35	7.37	7.39	0.14	0.41	0.54	0.27

**Table 3:** Fit of the ternary regression equation.

Predicted Depth (cm)	Ternary Regression Equation	*R* ^2^	RMSE
10	y_10_ = 1.403X_1_−0.065X_2_ − 0.313X_3_	0.915	2.1
40	y_40_ = 0.057X_1_ + 0.875X_2_ + 0.112X_3_	0.984	0.72
60	y_60_ = 0.093X_1_ + 0.008X_2_ + 0.684X_3_	0.976	0.27
70	y_70_ = 0.195X_1_ + 0.396X_2_ − 0.065X_3_	0.862	0.31
80	y_80_ = 0.187X_1_ + 0.413X_2_ − 0.0673X_3_	0.861	0.32
90	y_90_ = 0.196X_1_ − 0.413X_2_ − 0.068X_3_	0.865	0.32
100	y_100_ = 0.196X_1_ + 0.417X_2_ − 0.069X_3_	0.863	0.33
